# Low-dose esketamine for postoperative pain, sleep, and hemodynamic stability in functional endoscopic sinus surgery: a retrospective cohort study

**DOI:** 10.3389/fpain.2026.1858148

**Published:** 2026-08-11

**Authors:** Yan-Ping Zhao, Xun-Si Fu, Yan Jia, Feng Zhu, Li-Juan Shi

**Affiliations:** 1 Department of Anaesthesiology, The Affiliated Guangzhou Twelfth People's Hospital, Guangzhou Medical University, Guangzhou, China; 2 Central Laboratory, The Affiliated Guangzhou Twelfth People's Hospital, Guangzhou Medical University, Guangzhou, China

**Keywords:** esketamine, hemodynamic stability, postoperative pain, retrospective cohort study, sleep quality

## Abstract

**Objective:**

To evaluate the efficacy and safety of low-dose esketamine (0.2 mg/kg or 0.3 mg/kg) in improving postoperative pain, sleep quality, hemodynamic stability, and emotional status in functional endoscopic sinus surgery (FESS) patients.

**Methods:**

This retrospective cohort study analysed 135 chronic rhinosinusitis patients (January 2021–December 2023) divided into control (normal saline, *n* = 45), 0.2 mg/kg esketamine (*n* = 45), and 0.3 mg/kg esketamine (*n* = 45) groups. Propensity score matching (PSM)-balanced baselines. Primary outcome: 24-hour post-operative VAS pain score. Secondary outcomes included sleep/emotion/hemodynamic indices and adverse events. Python-based statistical analyses were performed.

**Results:**

After PSM, 40 patients/group were analysed. The 0.3 mg/kg (5.82 ± 1.89) and 0.2 mg/kg (6.53 ± 1.59) groups had significantly lower 24-hour VAS scores than the control group did (7.16 ± 0.93, *P* < 0.001), with the 0.3 mg/kg group being superior (*P* = 0.032). The eyesketamine group had better sleep quality (PSQI: 3.53 ± 0.69–6.31 ± 0.51 vs. 9.07 ± 1.12; *P* < 0.001), reduced anxiety/depression (HAM-A/HAM-D), improved resilience (CD-RISC), and less vasoactive drug use (*P* < 0.001). Adverse events were lower, with no increased neuropsychiatric reactions.

**Conclusion:**

The use of low-dose esketamine (particularly 0.3 mg/kg) in FESS patients is associated with clinically meaningful improvements in pain control, sleep quality, emotional well-being, and hemodynamic parameters, alongside a favorable safety profile with no increase in neuropsychiatric adverse events. These findings support its consideration as a viable non-opioid analgesic adjunct in this surgical setting.

## 1 Introduction

Chronic rhinosinusitis (CRS) is a common otolaryngological disease with a global prevalence of 5% to 15% that severely affects patients' quality of life and daily activities (1–4). Functional endoscopic sinus surgery (FESS) is the first-line surgical treatment for refractory CRS and removes diseased tissues while preserving normal nasal and sinus mucosal function (4). However, FESS is associated with postoperative pain, which is caused mainly by nasal packing-induced mucosal irritation and compression of nasal nerve endings and manifests as nasal distension, headache, and facial pain (5, 6). Even with routine analgesia, patients often experience moderate to severe pain (VAS score ≥5) within 24 h post-operatively, which further impairs sleep quality and induces anxiety or depression, delaying postoperative recovery (6).

Opioids are traditional perioperative analgesics but are associated with multiple adverse reactions, such as nausea, vomiting, hypoxemia, constipation, and central sensitization (7–12). With the global promotion of opioid-sparing anaesthesia, finding safe and effective nonopioid analgesic adjuvants has become a clinical research focus (3, 9). NSAIDs (nonsteroidal anti-inflammatory drugs) are a class of drugs with anti-inflammatory, analgesic and antipyretic effects. The main mechanism of action involves reducing the synthesis of prostaglandins by inhibiting cyclooxygenase (COX) in the body to reduce inflammatory reactions and pain. Although NSAIDs are very effective in the treatment of pain and inflammation, they may also cause some adverse reactions, such as gastrointestinal reactions, kidney damage, cardiovascular risk, blood system effects, allergic reactions, etc. Esketamine, the right-handed isomer of ketamine, is a unique intravenous anaesthetic with sedative, analgesic, and anaesthetic effects and mild respiratory depression (13, 14). Compared with ketamine, it has a 3–4-fold greater affinity for N-methyl-D-aspartate (NMDA) receptors, inhibiting central sensitization and reducing postoperative pain (13). Previous studies have shown that esketamine improves slow-wave sleep in gynecological surgery patients and alleviates postoperative anxiety and depression (15). However, its application in FESS patients remains underexplored, with no clear evidence on the optimal dosage and comprehensive efficacy (pain, sleep, mood, and hemodynamics) (16).

The purpose of this retrospective cohort study was to evaluate the efficacy and safety of two low doses of esketamine (0.2 mg/kg and 0.3 mg/kg) on postoperative pain, sleep quality, emotional state and hemodynamic stability by analysing the real clinical data of patients with FESS and to identify a safe and effective nonopioid analgesic method. The results of this study are expected to provide a practical nonopioid analgesic strategy for postoperative FESS management.

## 2 Materials and methods

### 2.1 Study design and ethical approval

This single-center retrospective cohort study was approved by the Ethics Committee of the Affiliated Guangzhou Twelfth People's Hospital (Approval No. 2021059). The study was conducted in accordance with the Declaration of Helsinki and Strengthening the Reporting of Observational Studies in Epidemiology (STROBE) guidelines. As this study represents a retrospective analysis of routine clinical data, the authors performed a secondary retrospective analysis of data derived from a prior prospective trial (such as an RCT) from a novel perspective, the ethics committee waived the requirement for additional informed consent. However, informed consent covering anesthesia, analgesia, and the use of patient data had already been obtained from all patients during their hospital stay.

### 2.2 Study population

The included patients were those who underwent FESS for CRS at the Affiliated Guangzhou Twelfth People's Hospital between January 2021–December 2023. The inclusion criteria were as follows: (1) age 18–70 years; (2) diagnosis of CRS (with or without nasal polyps) confirmed by clinical symptoms, nasal endoscopy, and computed tomography (CT); (3) American Society of Anesthesiologists (ASA) classification I-III; (4) complete clinical data and follow-up records (postoperative 1, 5, 9 days); and (5) cooperation with pain and sleep quality assessment. The exclusion criteria were as follows: (1) severe cardiovascular, respiratory, hepatic, or renal dysfunction; (2) a history of analgesic drug addiction, migraine, or psychiatric diseases (schizophrenia, epilepsy, etc.); (3) allergy to esketamine or other anaesthetic drugs used in the study; (4) a surgical duration <1 h or >3 h; (5) intraoperative blood loss >200 mL; and (6) severe postoperative infection or failure to complete follow-up.

### 2.3 Grouping criteria

Participants were assigned to groups through randomization based on a numbered table and single-blind procedures, and the criteria for group designation were based on medication records. A three-arm parallel-group design was employed, consisting of a control group, a 0.2 mg/kg group, and a 0.3 mg/kg group. The primary outcome was the 24-hour postoperative VAS pain score. Based on preliminary data, the minimal clinically important difference was predefined as a 2-point reduction, with a pooled standard deviation of 1.5, corresponding to an effect size (Cohen's f) of 0.67. At a significance level of *α* = 0.05% and 80% power, analysis of variance (ANOVA) indicated that 28 participants per group were required. Since three pairwise comparisons were planned, a Bonferroni correction (*α*' = 0.017) was applied to control the family-wise error rate, increasing the required sample size to 36–38 per group. Taking 36 as the baseline and accounting for a 20% dropout rate, we ultimately aimed to enroll 45 participants per group, for a total target sample size of 135. The control group received an intravenous injection of 3.0 mL of normal saline before anaesthesia induction; the 0.2 mg/kg group was given an intravenous injection of 0.2 mg/kg esketamine (dissolved in 3.0 mL of normal saline) before anaesthesia induction; and the 0.3 mg/kg group received an intravenous injection of 0.3 mg/kg esketamine (dissolved in 3.0 mL of normal saline) before anaesthesia induction. All other anaesthetic procedures were consistent among the groups: anaesthesia induction with propofol (1.0–2.0 mg/kg), sufentanil (0.5 μg/kg), and rocuronium (0.6 mg/kg) tracheal intubation under video laryngoscopy; anaesthesia maintenance with sevoflurane (1.0–3.0 VOL%); intermittent rocuronium; intraoperative BIS value maintained at 40–60; and target blood pressure reduced by 20% from baseline, with nitroglycerin or vasopressors used if necessary. Postoperative analgesia was standardized with flurbiprofen axetil 100 mg intravenously for all groups, with no additional opioids used.

### 2.4 Outcome measures

#### 2.4.1 Primary outcome

Visual Analogue Scale (VAS) pain score at 24 h (1 day) postoperatively: A 10-point scale (0 = no pain, 10 = severe pain), with scores of 0–3 (mild pain), 4–6 (moderate pain), and 7–10 (severe pain). Cronbach's *α* = 0.82–0.93, test–retest reliability = 0.91, validated for acute postoperative pain assessment (17). Secondary outcomes included (1) pain-related outcomes (VAS scores preoperatively and at postoperative days 5 and 9) and (2) sleep quality (PSQI scores) preoperatively and at postoperative days 1, 5, and 9. The PSQI includes 7 dimensions (subjective sleep quality, sleep latency, sleep duration, sleep efficiency, sleep disturbance, sleep medication use, and daytime dysfunction), with a total score of 0–21 (higher scores indicate poorer sleep quality); Cronbach's *α* = 0.83, split-half reliability = 0.79, and structural validity confirmed by confirmatory factor analysis (CFA) (18). (3) Emotional status: Hamilton Depression Scale (HAM-D, 24-item) and Hamilton Anxiety Scale (HAM-A) scores before surgery and at 1, 5, and 9 days after surgery. HAM-D scores: <8 (no depression), 8–20 (mild), 21–35 (moderate), and >35 (severe); HAM-A scores: <7 (no anxiety), 7–13 (possible anxiety), 14–20 (definite anxiety), 21–28 (significant anxiety), and ≥29 (severe anxiety). The Cronbach's *α* was 0.86, the interrater reliability was 0.88, and the criterion validity was validated against clinical diagnosis (19). (4) Psychological resilience: Connor-Davidson Resilience Scale (CD-RISC) scores preoperatively and at postoperative days 1, 5, and 9. The scale includes 25 items (5 dimensions), with a total score of 0–100 (higher scores indicate better resilience). Cronbach's *α* = 0.89, test–retest reliability = 0.87, and construct validity confirmed by exploratory factor analysis (EFA) (20). (5 Hemodynamic and vasoactive drug use: Intraoperative mean arterial pressure (MAP), heart rate (HR), total nitroglycerin usage (mgcases), and vasopressor usage (mgcases); incidence of intraoperative hypertensive/hypotensive events (hypertension: MAP >140/90 mmHg; hypotension: MAP <90/60 mmHg); incidence of intraoperative hypertension/hypotension events (hypertension: MAP>140/90 mmHg; hypotension: MAP<90/60 mmHg), or blood pressure fluctuations greater than 20% of baseline blood pressure. (6) Safety outcomes: Incidence of postoperative adverse events within 9 days, including general adverse events (hypoxemia, nausea/vomiting, pruritus, hypertension, hypotension) and esketamine-specific neuropsychiatric adverse events (hallucinations, nightmares, dissociation). Adverse events were recorded via structured interviews at postoperative days 1, 5, and 9.

### 2.5 Data collection

This study is a retrospective reanalysis of data from a previous prospective trial (e.g., RCT) conducted by our team, carried out from a different perspective or for a different purpose. Clinical data were extracted from the hospital's electronic medical records system and anaesthesia information management system, including (1) baseline data [age, sex, BMI, underlying diseases (hypertension, diabetes)], ASA classification, disease course, surgical time, anaesthesia time, and recovery time; (2) outcome indicators (VAS, PSQI, HAM-D, HAM-A, and CD-RISC at each time point); (3) intraoperative data (MAP, HR, nitroglycerin/vasopressor usage, and adverse events); and (4) postoperative adverse event records. In order to reduce potential information bias, anesthesia procedures, postoperative interviews, and scale-based assessments were performed by separate personnel. Two independent researchers verified the data accuracy, with discrepancies resolved by discussion with a senior anesthesiologist. Nonetheless, because the primary endpoints—VAS, HAM-A, HAM-D, and PSQI—are subjective in nature, we cannot completely rule out the effects of placebo and expectancy bias from both patients and caregivers. This remains a significant limitation of our study.

### 2.6 Statistical analysis

#### 2.6.1 Data preprocessing and propensity score matching (PSM)

Data preprocessing and statistical analysis were performed using Python 3.9 (Python Software Foundation, Wilmington, DE, USA) with libraries including pandas (1.5.3), numpy (1.24.3), scikit-learn (1.2.2), scipy (1.10.1), and matplotlib (3.7.1). Missing data were handled by multiple imputations (≤ 5% missing rate). To address the inherent limitations of propensity score matching (PSM), we performed balance diagnostics. PSM was used to balance baseline confounding factors between groups: Age, sex, BMI, ASA classification, disease course, and surgical time were included in the logistic regression model to calculate propensity scores; 1:1:1 matching was performed with a calliper width of 0.02; and a standardized mean difference (SMD) < 0.1 was considered to indicate balanced baseline characteristics.

#### 2.6.2 Statistical methods

Normally distributed continuous data are expressed as the mean ± standard deviation ($\bar{x}$±s), and comparisons between groups were performed using one-way analysis of variance (ANOVA) followed by Bonferroni correction for multiple comparisons; skewed distributed continuous data are expressed as the median (interquartile range) [M(IQR)], with group comparisons conducted via the Kruskal‒Wallis H test; repeated measures data (including VAS, PSQI, HAM-D scores and other indicators at multiple time points) were analysed using repeated-measures ANOVA, with Greenhouse–Geisser correction applied for sphericity violation; and categorical data are presented as counts (percentages) [n(%)], and comparisons were performed using the *χ*² test or Fisher's exact test as appropriate; the Pearson correlation coefficient was used to conduct correlation analysis between pain and sleep quality; statistical significance was set at *P* < 0.05. *post-hoc* power analyses of secondary outcomes were performed using G*Power.

## 3 Results

### 3.1 Study population and baseline characteristics

A total of 158 CRS patients who underwent FESS were initially screened; 52 patients were excluded, and 135 patients were included in the initial retrospective analysis. After PSM, 40 patients in each group (120 total) were included in the final analysis, with no significant differences in baseline characteristics between groups (all SMD <0.1, *P* > 0.05) (Table 1).

**Table 1:** Baseline characteristics of patients before and after propensity score matching.

Characteristic	Total (*n* = 135)	Control (*n* = 45)	0.2 mg/kg esketamine (*n* = 45)	0.3 mg/kg esketamine (*n* = 45)	*P*-value	SMD
Gender (male/female)	76/59	25/20	32/13	29/16	0.306	0.08
Age(years, $\bar{x}$±s)	42.07 ± 13.19	43.67 ± 13.49	41.58 ± 12.81	40.96 ± 13.27	0.595	0.05
BMI (kg/m², $\bar{x}$±s)	23.40 ± 2.98	23.22 ± 3.04	23.25 ± 3.04	23.74 ± 2.85	0.652	0.03
Underlying diseases, *n* (%)					0.347	0.06
Hypertension	10 (7.4)	5 (11.1)	3 (6.7)	2 (4.4)		
Diabetes	3 (2.2)	2 (4.4)	1 (2.2)	0 (0.0)		
ASA classification, *n* (%)					0.574	0.07
I	108 (80.0)	36 (80.0)	34 (75.6)	38 (84.4)		
II	27 (20.0)	9 (20.0)	11 (24.4)	7 (15.6)		
Surgical time (min, $\bar{x}$±s)	116.50 ± 30.64	119.80 ± 32.96	112.82 ± 29.70	116.89 ± 29.26	0.557	0.04
Anaesthesia time (min, $\bar{x}$±s)	182.33 ± 34.32	182.89 ± 35.77	179.27 ± 32.43	184.84 ± 34.77	0.737	0.06
Disease course (years, $\bar{x}$±s)	5.13 ± 6.71	5.93 ± 7.76	4.69 ± 5.18	4.76 ± 7.20	0.622	0.05

### 3.2 Primary outcome: postoperative 24-hour VAS pain score

After PSM, the VAS score at 24 h post-operatively was significantly lower in the 0.3 mg/kg group (5.82 ± 1.89) and 0.2 mg/kg group (6.53 ± 1.59) than in the control group (7.16 ± 0.93) (F = 17.28; *P* < 0.001). Pairwise comparisons revealed significant differences between the 0.3 mg/kg group and the control group (*P* < 0.001), between the 0.2 mg/kg group and the control group (*P* = 0.012), and between the 0.3 mg/kg group and the 0.2 mg/kg group (*P* = 0.032) (Table 2).

**Table 2:** Comparison of VAS scores among the three groups ($\bar{x}$±s, points).

Time Point	Control (*n* = 40)	0.2 mg/kg esketamine (*n* = 40)	0.3 mg/kg esketamine (*n* = 40)	F-value	*P*-value
Preoperative	7.38 ± 1.17	7.22 ± 1.49	6.76 ± 1.53	1.21	0.302
Postoperative 1 day	7.16 ± 0.93	6.53 ± 1.59	5.82 ± 1.89	17.28	<0.001
Postoperative 5 days	5.11 ± 0.75	4.67 ± 0.77	3.69 ± 1.73	28.45	<0.001
Postoperative 9 days	3.56 ± 0.59	2.56 ± 0.55	1.47 ± 0.55	126.71	<0.001

### 3.3 Secondary outcomes

#### 3.3.1 Pain scores (VAS) at different time points

Repeated-measures ANOVA revealed significant main effects of group (F = 156.52; *P* < 0.001), time (F = 8.65; *P* < 0.001), and group × time interaction (F = 23.41; *P* < 0.001). VAS scores decreased gradually over time in all groups, with the esketamine groups having significantly lower scores than the control group did at postoperative days 5 and 9 (all *P* < 0.05), and the 0.3 mg/kg group had the lowest scores (Table 2). *post-hoc* power analyses of *f* = 0.54–0.55 shows that the dose-*vs*-dose power is estimated in the range of 0.45–0.60, which is below the conventional threshold of 0.80.

#### 3.3.2 Sleep quality (PSQI score)

Repeated-measures ANOVA revealed significant main effects of group (F = 518.73; *P* < 0.001), time (F = 103.41; *P* < 0.001), and group × time interaction (F = 45.62; *P* < 0.001). The PSQI scores at postoperative days 1, 5, and 9 were significantly lower in the esketamine groups than in the control group (all *P* < 0.001), with the 0.3 mg/kg group showing the best sleep quality (Table 3). Correlation analysis revealed a significant positive correlation between VAS scores and PSQI scores at postoperative day 1 (r = 0.72, *P* < 0.001), indicating that pain relief was associated with improved sleep quality. *post-hoc* power analyses of PSQI shows an exceptionally massive effect (equivalent to *f* > 2.0). Even with a small sample size and multiple groups, the power to detect any difference between doses exceeds **0.99**.

**Table 3:** Comparison of PSQI scores among the three groups ($\bar{x}$±s, points).

Time Point	Control (*n* = 40)	0.2 mg/kg esketamine (*n* = 40)	0.3 mg/kg esketamine (*n* = 40)	F-value	*P*-value
Preoperative	17.67 ± 2.24	18.02 ± 2.46	16.76 ± 2.34	2.15	0.121
Postoperative 1 day	15.32 ± 1.87	11.25 ± 1.63	7.89 ± 1.45	289.64	<0.001
Postoperative 5 days	9.07 ± 1.12	6.31 ± 0.51	3.53 ± 0.69	518.73	<0.001
Postoperative 9 days	7.21 ± 0.98	4.15 ± 0.76	2.89 ± 0.58	426.38	<0.001

#### 3.3.3 Emotional Status (HAM-D and HAM-A scores)

For HAM-D scores, repeated-measures ANOVA revealed significant main effects of group (F = 4.28; *P* = 0.009), time (F = 13.93; *P* < 0.001), and group × time interaction (F = 14.32; *P* < 0.001). On postoperative day 9, the HAM-D scores in both esketamine groups were significantly lower than those in the control group (all *P* < 0.05), while no significant difference was observed between the two esketamine groups on postoperative day 9 (*P* = 0.612) (Table 4). *post-hoc* power analysis shows a medium effect combined with a strict *α* = 0.0167 and a multi-group dose comparison yields power well below 0.20. This is the most critically underpowered analysis in the study.

**Table 4:** Comparison of HAM-D and HAM-A scores among the three groups (x ± s, points).

Index	Time Point	Control (*n* = 40)	0.2 mg/kg esketamine (*n* = 40)	0.3 mg/kg esketamine (*n* = 40)	F-group	*P*-group
HAM-D	Preoperative	21.64 ± 9.93	20.56 ± 9.78	21.93 ± 10.65	0.32	0.729
	Postoperative 1 day	20.07 ± 9.34	19.76 ± 9.34	19.42 ± 9.93	0.15	0.861
	Postoperative 5 day	10.93 ± 5.81	12.29 ± 6.31	13.11 ± 5.96	1.87	0.158
	Postoperative 9 day	11.73 ± 4.73	9.64 ± 3.83	9.44 ± 4.83	4.52	0.013
HAM-A	Preoperative	24.47 ± 4.85	24.71 ± 5.12	23.56 ± 3.97	0.48	0.621
	Postoperative 1 day	24.96 ± 4.26	21.98 ± 4.28	22.00 ± 2.48	8.76	<0.001
	Postoperative 5 day	19.42 ± 9.94	18.51 ± 2.81	18.31 ± 2.89	0.35	0.706
	Postoperative 9 day	19.29 ± 3.53	17.53 ± 2.83	14.89 ± 3.17	16.89	<0.001

For HAM-A scores, repeated-measures ANOVA demonstrated significant main effects of group (F = 17.48, *P* < 0.001), time (F = 9.48, *P* = 0.004), and group × time interaction (F = 17.31, *P* < 0.001). On postoperative days 1, 5, and 9, HAM-A scores were significantly lower in the esketamine groups than in the control group (all *P* < 0.05), with the 0.3 mg/kg esketamine group having the lowest scores on postoperative day 9 (14.89 ± 3.17) (Table 4). *post-hoc* power analyses of HAM-A for *f* = 0.54–0.55 shows that the dose-vs-dose power is estimated in the 0.45–0.60 range, which is below the conventional 0.80 threshold.

#### 3.3.4 Psychological resilience (CD-RISC scores)

Repeated-measures ANOVA revealed significant group (F = 28.74; *P* < 0.001), time (F = 55.15; *P* < 0.001), and group × time interaction (F = 34.09; *P* < 0.001) effects. CD-RISC scores increased gradually over time in all groups, with the esketamine groups having significantly higher scores than the control group did at postoperative days 1, 5, and 9 (all *P* < 0.001), and the 0.3 mg/kg group had the highest scores (Table 5). *post-hoc* power analysis shows that the uncorrected alpha and large effect size provide a solid foundation. However, because the dose comparison involves smaller subgroups and a more complex contrast (e.g., linear trend or pairwise), the power is estimated in the **0.70–0.85** range—moderate to good, but not outstanding.

**Table 5:** Comparison of CD-RISC scores among the three groups ($\bar{x}$±s, points).

Time Point	Control (*n* = 40)	0.2 mg/kg esketamine (*n* = 40)	0.3 mg/kg esketamine (*n* = 40)	F-value	*P*-value
Preoperative	43.31 ± 9.45	41.87 ± 9.42	40.87 ± 7.90	0.56	0.572
Postoperative 1 day	46.04 ± 7.10	47.36 ± 11.98	53.16 ± 8.94	6.89	0.001
Postoperative 5 day	54.40 ± 10.40	66.47 ± 8.15	74.49 ± 8.70	58.76	<0.001
Postoperative 9 day	61.51 ± 11.35	73.38 ± 12.24	82.71 ± 12.98	72.45	<0.001

#### 3.3.5 Traoperative hemodynamics and vasoactive drug use

Intraoperative MAP and HR fluctuations were significantly smaller in the esketamine groups than in the control group (*P* < 0.05). The control group had higher levels of nitroglycerin and vasopressor use, as well as higher rates of hypertension and hypotension (all *P* < 0.05) (Table 6). This dose-dependent reduction in the reliance on vasoactive drugs further supports the role of esketamine in optimizing perioperative hemodynamic management. *post-hoc* power calculations were performed for the key hemodynamic parameters using the observed effect sizes (partial *η*² from F-values). The achieved powers for MAP fluctuation (F = 42.15) and nitroglycerin use (F = 89.64) were both above 0.95, demonstrating that our per-group sample size of 40 was sufficient to identify the observed effects.

**Table 6:** Comparison of intraoperative vasoactive drug use and adverse events [$\bar{x}$±s or n(%)].

Index	Control (*n* = 40)	0.2 mg/kg esketamine (*n* = 40)	0.3 mg/kg esketamine (*n* = 40)	Statistic	*P*-value
Nitroglycerin usage (cases, $\bar{x}$±s)	7 ± 2	3 ± 1	1.0 ± 1.0	F = 89.64	<0.001
Vasopressor usage (cases, $\bar{x}$±s)	3 ± 1	0.0 ± 0.0	2 ± 1	F = 67.89	<0.001
Hypertensive events, n(%)	9 (22.5)	3 (7.5)	0 (0.0)	*χ*²=12.67	0.002
Hypotensive events, n(%)	3 (7.5)	0 (0.0)	0 (0.0)	Fisher's exact	0.023
MAP fluctuation (mmHg, $\bar{x}$±s)	12.8 ± 3.5	7.2 ± 2.1	5.9 ± 1.8	F = 42.15	<0.001
HR fluctuation (bpm, $\bar{x}$±s)	10.5 ± 2.8	5.6 ± 1.9	4.8 ± 1.5	F = 38.62	<0.001

### 3.4 Postoperative adverse events

The total incidence of postoperative adverse events was significantly lower in the esketamine group than in the control group (*χ*²=18.96; *P* < 0.001). Specifically, the incidence of nausea/vomiting (20.0% vs. 0.0% vs. 2.5%, *P* < 0.001), hypertension (22.5% vs. 7.5% vs. 0.0%, *P* = 0.002), and hypotension (7.5% vs. 0.0% vs. 0.0%, *P* = 0.023) was significantly lower in the esketamine group. There were no significant differences in hypoxemia or pruritus. Esketamine-specific neuropsychiatric adverse events were rare: 1 case (2.5%) of nightmares in the 0.3 mg/kg group and no nightmares or hallucinations or dissociation in any group (Table 7). *post-hoc* power analyses based on the observed effect sizes, the achieved powers for nausea/vomiting (Fisher's exact test, *P* = 0.003) and hypertension (*χ*²=12.67, *P* = 0.002) were 0.82 and 0.89, respectively, indicating that our sample size was sufficient to detect significant differences in these events. For adverse events without significant differences (e.g., hypoxemia and pruritus), the *post-hoc* powers were relatively low (0.31 and 0.45, respectively), suggesting that these null findings should be interpreted with caution and warrant validation in larger cohorts.

**Table 7:** Comparison of postoperative adverse events among the three groups [n (%)].

Adverse Event	Control (*n* = 40)	0.2 mg/kg Esketamine (*n* = 40)	0.3 mg/kg Esketamine (*n* = 40)	χ²/Fisher's exact	*P*-value
Hypoxemia	4 (10.0)	1 (2.5)	2 (5.0)	Fisher's exact	0.187
Nausea & Vomiting	8 (20.0)	0 (0.0)	1 (2.5)	Fisher's exact	0.003
Pruritus	5 (12.5)	2 (5.0)	1 (2.5)	χ²=4.89	0.086
Hypertension	9 (22.5)	3 (7.5)	0 (0.0)	χ²=12.67	0.002
Hypotension	3 (7.5)	0 (0.0)	0 (0.0)	Fisher's exact	0.023
Nightmares	0 (0.0)	0 (0.0)	0 (0.0)	Fisher's exact	0.315
Hallucinations	0 (0.0)	0 (0.0)	0 (0.0)	-	-
Dissociation	0 (0.0)	0(0.0)	0(0.0)	-	-

## 4 Discussion

Although this study is a single-center retrospective cohort design, and despite the use of propensity score matching (PSM) to control for confounding factors, selection bias and recall bias have not been completely eliminated. Nevertheless, our data strongly suggest that low-dose esketamine (0.2 mg/kg and 0.3 mg/kg) confers significant clinical benefits in improving postoperative pain, sleep quality, emotional status, and hemodynamic stability in patients undergoing FESS, while demonstrating a favorable safety. The primary outcome showed that 0.3 mg/kg esketamine provided the best analgesic effect at 24 h post-operatively, which is consistent with the dose‒response relationship observed in the secondary outcomes.

### 4.1 Efficacy of low-dose esketamine in postoperative pain relief

Postoperative pain is primarily driven by mechanical irritation from nasal packing and local inflammatory responses (21, 22). In this study, both doses of esketamine were associated with significantly reduced VAS scores at 24 h postoperatively, with the 0.3 mg/kg group achieving a clinically meaningful reduction in pain (≥1.3 points vs. control). Although the prespecified target of a 2-point decrease was not met, previous evidence from Myles et al. suggests that a 10-mm change on a 100-mm VAS represents a clinically important difference (23). These analgesic effects are attributable to esketamine's unique mechanism of action, which includes N-methyl-D-aspartate (NMDA) receptor blockade to inhibit central sensitization, attenuation of opioid-induced hyperalgesia, and enhancement of incisional analgesia (24, 25). Unlike opioids, esketamine does not cause respiratory depression or constipation, making it a safer alternative for postoperative analgesia in patients undergoing functional endoscopic sinus surgery (FESS). Notably, the analgesic benefit of esketamine may persist for several days after surgery, likely due to the sustained activity of its active metabolite, norketamine (elimination half-life 2–4 h) (26), which aligns well with the analgesic requirements following FESS.

### 4.2 Improvement in sleep quality and emotional status

Postoperative pain during FESS and nasal packing-induced ventilation dysfunction often lead to poor sleep quality, which further exacerbates anxiety and depression (27, 28).Considering the short elimination half-life of esketamine, it is plausible that the low-dose infusion given during surgery predominantly acts on acute postoperative pain. The consequent pain relief may, in turn, secondarily contribute to better mood and enhanced sleep quality, as opposed to a direct and prolonged pharmacological effect of the drug itself. It was shown that esketamine significantly reduces the incidence of postoperative sleep disturbance on the first day after surgery (29). Our study found a significant association between esketamine administration and reduced PSQI scores on postoperative day 5, with the 0.3 mg/kg group achieving a PSQI score of 3.53 ± 0.69, indicating excellent sleep quality. Correlation analysis revealed a positive association between pain relief and improved sleep quality (r = 0.72), suggesting that pain alleviation may partially mediate the relationship between esketamine and sleep improvement. Furthermore, esketamine reduced HAM-D and HAM-A scores, with more pronounced effects observed on postoperative day 9, which may be attributed to its antidepressant and anxiolytic properties. Mechanistically, esketamine acts as an NMDA receptor antagonist, promoting the release of neurotrophic factors such as BDNF, thereby enhancing neuroplasticity and synaptogenesis (28, 29). The concurrent improvement in pain, sleep, and emotional status is particularly beneficial for FESS patients, as it may facilitate faster postoperative recovery and improve overall quality of life.

### 4.3 Hemodynamic stability and safety

Maintaining perioperative hemodynamic stability is critical for FESS patients, particularly in those with underlying cardiovascular disease. In the present study, patients receiving esketamine demonstrated reduced intraoperative variability in MAP and HR, lower cumulative requirements for nitroglycerin and vasopressors, and fewer episodes of hypertension and hypotension relative to the control cohort. These observations are in keeping with prior evidence that esketamine, when co-administered with propofol, may dampen baroreflex sensitivity and thereby promote hemodynamic equilibrium (30).In terms of safety, the esketamine groups experienced a reduced frequency of common adverse events, including hypoxemia, nausea/vomiting, and hypertension, versus the control group. Notably, esketamine-associated neuropsychiatric effects—hallucinations, nightmares, and dissociative symptoms—were infrequent, with merely one instance of nightmare noted in the 0.3 mg/kg group. This reassuring safety profile is likely related to the low-dose regimen adopted (0.2–0.3 mg/kg) and the absence of preexisting psychiatric conditions among enrolled patients (31, 32). Taken together, these data support the safety and tolerability of low-dose esketamine in the FESS setting.

### 4.4 Novelty and clinical implications

The novelty of this study lies in the following: (1) It is the first retrospective cohort study to evaluate the comprehensive efficacy of low-dose esketamine in FESS patients, targeting the unique pathological chain of “pain-sleep disturbance-anxiety/depression” caused by nasal packing; (2) It clarifies the optimal dose (0.3 mg/kg) of esketamine for FESS postoperative management, filling the gap in clinical dosage guidance; and (3) it provides real-world evidence for opioid-sparing anaesthesia in otolaryngological surgery, which aligns with global clinical trends.

Clinically, our findings suggest that low-dose esketamine (0.3 mg/kg) can be used as a safe and effective nonopioid analgesic adjuvant for FESS patients, especially those at high risk of adverse opioid-related events or poor sleep quality. A standardized postoperative analgesia protocol (NSAIDs＋esketamine) can be widely applied in clinical practice to improve patient outcomes.

### 4.5 Limitations

This study has several limitations: (1) It is a single-center retrospective cohort study, although propensity score matching (PSM) was employed, selection bias and recall bias could not be completely eliminated; (2) the follow-up period was limited to 9 days, and long-term outcomes (e.g., 1-month sleep quality and recurrence of pain) were not evaluated; (3) the sample size was relatively small (45 patients per group), and multicenter studies with larger sample sizes are needed to validate the findings; and (4) we did not evaluate the effect of esketamine on nasal mucosal healing, which warrants further research.(5)The relatively small sample size was listed as one of the limitations of this study.

## 5 Conclusion

Low-dose esketamine, particularly at 0.3 mg/kg, effectively alleviates postoperative pain, improves sleep quality, reduces anxiety and depression, stabilizes perioperative hemodynamics, and decreases the incidence of adverse events in patients undergoing FESS. These findings support its use as a safe, practical, non-opioid analgesic strategy for postoperative FESS management, with considerable potential for clinical application.

## Data Availability

The raw data supporting the conclusions of this article will be made available by the authors, without undue reservation.
